# Morphology and Phylogenetics of Benthic *Prorocentrum* Species (Dinophyceae) from Tropical Northwestern Australia

**DOI:** 10.3390/toxins11100571

**Published:** 2019-09-30

**Authors:** Arjun Verma, Aniuska Kazandjian, Chowdhury Sarowar, D. Tim Harwood, J. Sam Murray, Insa Pargmann, Mona Hoppenrath, Shauna A. Murray

**Affiliations:** 1 Climate Change Cluster, University of Technology Sydney, Ultimo, NSW 2007, Australia; akazandj@gmail.com; 2 Laboratorio de Sistemática Molecular, Centro de Biodiversidad Marina, Universidad Simón Bolívar, Caracas 89000, Venezuela; 3 Sydney Institute of Marine Sciences, Mosman, NSW 2088, Australia; Chowdhury.Sarowar@sims.org.au; 4 Seafood Safety Research Programme, Cawthron Institute, Nelson 7010, New Zealand; tim.harwood@cawthron.org.nz (D.T.H.); Sam.Murray@cawthron.org.nz (J.S.M.); 5 Institut für Biologie und Umweltwissenschaften, Carl von Ossietzky Universität Oldenburg, D-26129 Oldenburg; Germany; Insel-pinsel@web.de (I.P.); mhoppenrath@senckenberg.de (M.H.); 6 Senckenberg am Meer, German Centre for Marine Biodiversity Research (DZMB), D-26382 Wilhelmshaven, Germany

**Keywords:** benthic dinoflagellates, phylogeny, *Prorocentrum*, taxonomy

## Abstract

Approximately 70 species of *Prorocentrum* are known, of which around 30 species are associated with benthic habitats. Some produce okadaic acid (OA), dinophysistoxin (DTX) and their derivatives, which are involved in diarrhetic shellfish poisoning. In this study, we isolated and characterized *Prorocentrum concavum* and *P. malayense* from Broome in north Western Australia using light and scanning electron microscopy as well as molecular sequences of large subunit regions of ribosomal DNA, marking the first record of these species from Australian waters. The morphology of the motile cells of *P. malayense* was similar to *P. concavum* in the light microscopy, but differed by the smooth thecal surface, the pore pattern and the production of mucous stalk-like structures and a hyaline sheath around the non-motile cells. *P. malayense* could also be differentiated from other closely related species, *P. leve* and *P. foraminosum*, despite the similarity in thecal surface and pore pattern, by its platelet formula and morphologies. We tested the production of OA and DTXs from both species, but found that they did not produce detectable levels of these toxins in the given culturing conditions. This study aids in establishing more effective monitoring of potential harmful algal taxa in Australian waters for aquaculture and recreational purposes.

## 1. Introduction

In recent decades, large-scale biodiversity surveys and high-throughput metabarcoding studies of coastal and oceanic environments have shown that approximately 8–10 times more operational taxonomic units (OTUs) of microbial eukaryotes are present than the number of previously described species, with approximately half of 18S rDNA richness made up of dinoflagellate (Dinophyceae, Alveolata) sequences [1,2,3]. Studies on marine sediments have highlighted diverse and abundant assemblages of dinoflagellates that play vital roles in benthic ecosystems but remain poorly understood [2,4,5,6,7]. Certain benthic dinoflagellates are known to produce potent toxic molecules that adversely affect aquaculture, fisheries and human health [8,9,10,11]. Among them, species of the genus *Prorocentrum* C.G.Ehrenberg are recurrently reported from tropical and temperate waters, where they occur in benthic, epibenthic and planktonic habitats [5,12]. Approximately 70 species of *Prorocentrum* are known, with *Prorocentrum micans* C.G.Ehrenberg as the type, of which around 30 species are associated with sediments and live epiphytically on macroalgal surfaces, floating detritus and corals [5,12,13,14].

Morphologically, prorocentroid dinoflagellates are quite distinct compared to other dinoflagellates. They have two larger thecal plates separated by a sagittal suture and tiny platelets in the periflagellar area [12,13]. They are also devoid of the typical dinoflagellate cingulum and sulcus but possess two typical dinoflagellate flagella arising from the flagellar pore [12,15]. The taxonomy of *Prorocentrum* is based mainly on criteria such as cell shape and size, thecal plate surface morphology (ornamentation and pore patterns), intercalary band morphology and the architectural details of the periflagellar area (number of platelets, spines, collars and protrusions) [12]. Ultrastructural features such as the presence of trichocysts and mucocysts and the presence or organization of pyrenoids in the plastids have also been used for taxonomic descriptions [12]. However, the existence of cryptic species, brief descriptions of type specimens and conflicting morphological reports based on light and scanning microscopy have caused difficulty in identifying and distinguishing *Prorocentrum* species, similar to other benthic dinoflagellate taxa [5,12,16,17,18,19,20,21,22]. Novel sampling tools and the increased use of molecular techniques have enabled the revision of taxonomic issues in numerous taxa by enabling differentiation between cryptic and pseudo-cryptic species, as well as the description of new species [17,18,23].

Several *Prorocentrum* species, namely *P. lima* (Ehrenberg) Stein; *P. cordatum* (Ostenfeld) Dodge (as *P. minimum* (Pavillard) Schiller); *P. borbonicum* Ten-Hage, Turquet, Quod, Puiseux-Dao and Couté; *P. concavum* Fukuyo; *P. leve* Faust, Kibler, Vandersea, Tester and Litaker; *P. rhathymum* A.R. Loeblich III, Sherley and Schmidt; *P. hoffmannianum* Faust emend. Fraga (also *P. maculosum* Faust); and *P. caipirignum* Fraga, Menezes and Nascimento have been shown to produce complex toxic molecules such as okadaic acid (OA); dinophysistoxin (DTX)-1, -2, -4 and -5c; borbotoxins; and prorocentrolides [24,25,26,27,28,29,30,31,32,33,34,35,36]. OA and/or its analogues are complex lipid- and water- soluble polyether molecules that have been associated with diarrhetic shellfish poisoning (DSP) in humans due to the consumption of contaminated clams, crabs and mussels [37,38].

**Figure 1:**
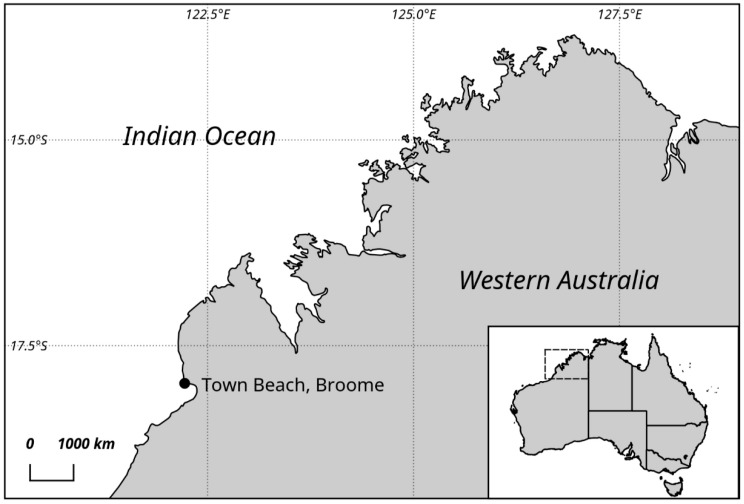
Map of the north Western coastline of Australia, showing the sampling location.

Increasing reports of novel potentially toxic *Prorocentrum* species from tropical and temperate waters, as well as the expanding shellfish aquaculture industry in northern Australia, suggest the need for baseline data on their diversity and distribution such that appropriate seafood safety monitoring can be established. In this study, we report on the morphological and phylogenetic characterization of *Prorocentrum* species based on samples collected from a benthic ecosystem in tropical north Western Australia (Figure 1) using a large ribosomal subunit gene (LSU rDNA), toxicological analysis and light and scanning electron microscopy. A recently described *Prorocentrum* species, *P. malayense* Z.F.Lim, Leaw and P.T.Lim, was reported, along with the closely related *P. concavum*. The strains were tested for the production of OA and DTXs using liquid chromatography–mass spectrometry (LC–MS/MS) analysis.

## 2. Results

### 2.1. Morphology of Prorocentrum Species

#### 2.1.1. Prorocentrum Concavum

**Figure 2:**
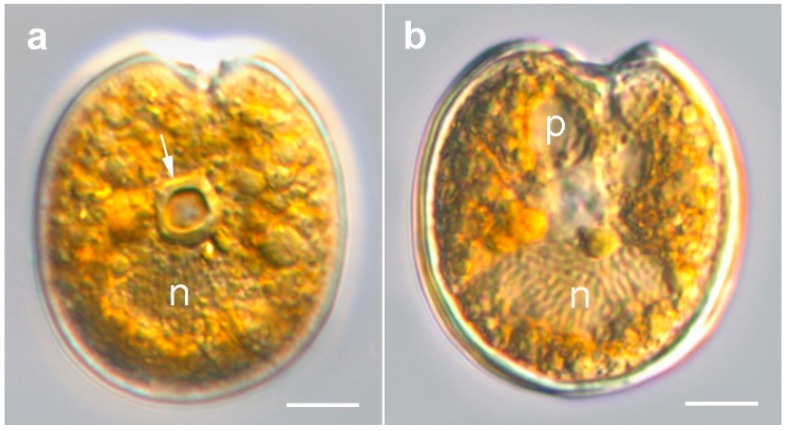
Light micrographs of *Prorocentrum concavum* BRM1. (**a**) Right lateral view showing the central pyrenoid (arrow) with a starch sheath (ring-like structure) and the posterior nucleus (n). (**b**) Mid cell focus: note the pusule (p) and nucleus (n). Scale bars = 10 µM.

**Figure 3:**
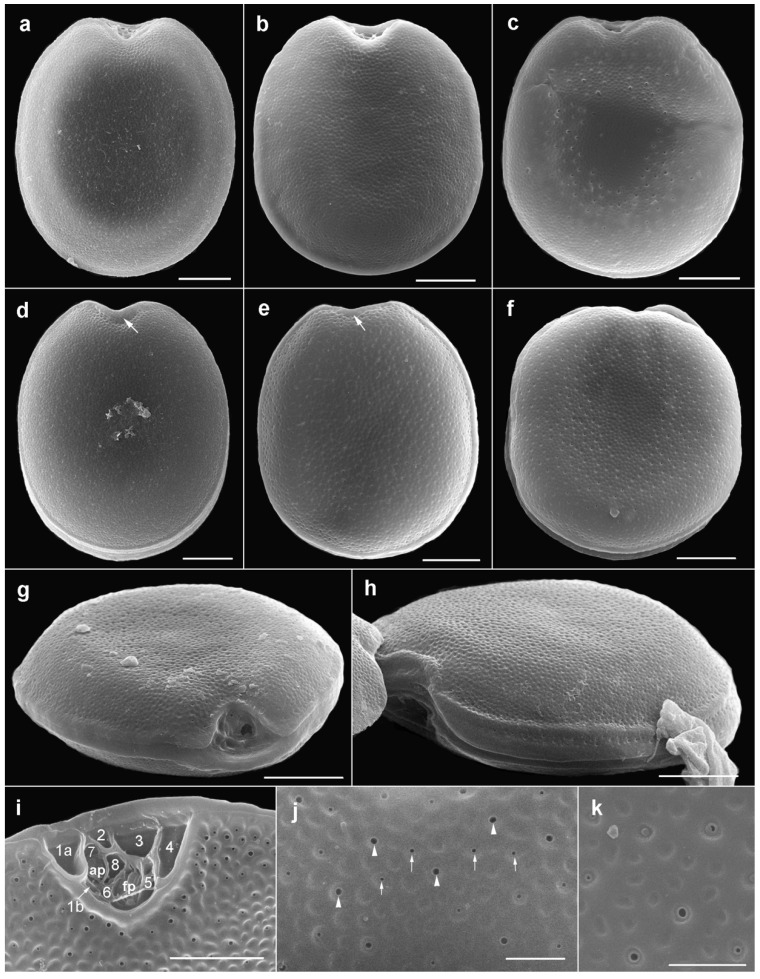
Scanning electron micrographs of *Prorocentrum concavum* BRM1. (**a**–**c**) Right lateral views. (**d**–**f**) Left lateral views: note the apical collar (arrow). (**g**) Apical to right lateral view showing the periflagellar area and the lateral flattening. (**h**) Ventral to left lateral view showing the intercalary band and the cell flattening. (**i**) Platelets in the periflagellar area. (**j**,**k**) Detail of thecal surface: note the large (arrowheads) and small (arrows) pores. Scale bars represent 10 μM in (**a**–**h**), 5 μM in (**i**), and 2 μM in (**j**,**k**).

Description: Cells were broad oval, 40.6–50.2-μM long and 36.2–46.3-μM deep (dorsoventral width; *n* = 16) (Figure 2a,b and Figure 3a–f). The golden-brown chloroplasts had a central pyrenoid visible due to the starch sheath (appearing as a ring-like structure) (Figure 2a). The large oval nucleus was located posterior and a pusule was sometimes visible anterior (Figure 3a,b). The periflagellar area was wide and V-shaped (Figure 2a and Figure 3a–c,g,i), slightly excavating the right thecal plate (Figure 3a–c), and had a more or less developed collar on the left thecal plate (Figure 3d,e). Both lateral sides were slightly concave to flat (Figure 3a–h). The intercalary bands were smooth (Figure 3g,h). Thecal plates had a reticulate-foveate ornamentation of variable intensity (Figure 3a–h). Thecal pores of two size classes (Figure 3i–k) were scattered over the thecal surface (located always inside the depressions) with the plate centre devoid of pores (Figure 2b,c,f), and pores become a bit denser close to the margin (Figure 3f,h,i), especially in the apical area. No marginal row of pores was recognized. Large pores were 0.15–0.25 μM, and small pores 0.05–0.10 μM in diameter. Pores were encircled by a ring-like structure (Figure 3i–k). Nine platelets were present in the periflagellar area—1a, b, 2, 3, 4, 5, 6, 7 and 8—labelled according to the system proposed by Hoppenrath et al. [12] (Figure 3i). Platelet 1a had no special ornamentation or structure, but maybe a slightly raised rim. The second platelet was relatively small, and the fourth platelet was elongated.

#### 2.1.2. Prorocentrum Malayense

**Figure 4:**
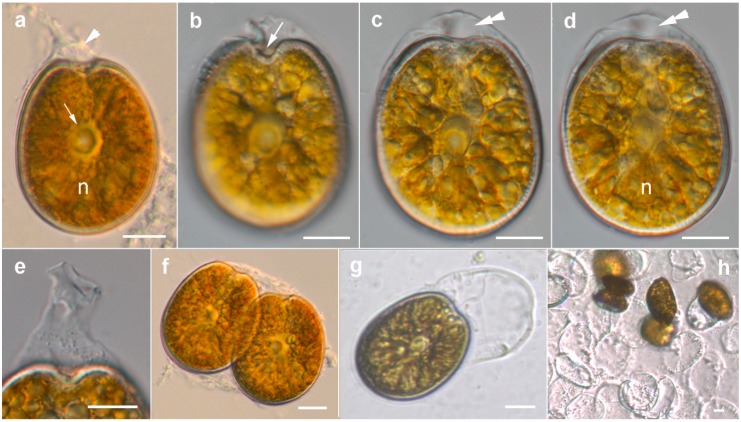
Light micrographs of *Prorocentrum malayense* BRM2. (**a**) Right lateral view showing the central pyrenoid (arrow) with a starch sheath (ring-like structure) and the posterior nucleus (n). Note the irregular mucous stalk-like structures at the apical cell end (arrowhead); (**b**–**d**) the same cell in different focal planes. Note the periflagellar area (arrow), the skin-like sheath (double arrowhead) and the posterior nucleus (n); (**e**) irregular mucous stalk-like structures at the apical cell end; (**f**) cell division inside a hyaline mucus cover; (**g**) cell leaving its skin-like sheath; (**h**) disembodied remains of the sheaths. Scale bars = 10 μM.

**Figure 5:**
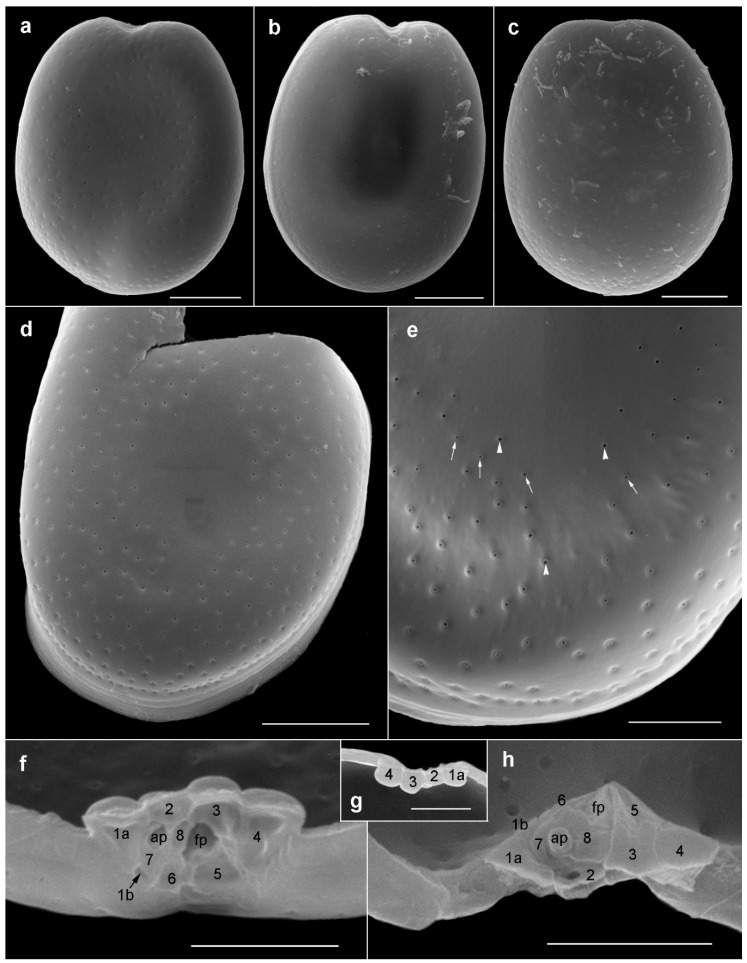
Scanning electron micrographs of *Prorocentrum malayense* BRM2. (**a**,**b**) Right lateral views. (**c**) Left lateral view. (**d**,**e**) Details of thecal surface: note the large (arrowheads) and small (arrows) pores. (**f**–**h**) Platelets in the periflagellar area. (**f**) Outside view. (**g**) View of the sagittal suture plane of the right thecal plate. (**h**) Inside view. Scale bars represent 10 μM in (**a**–**d**) and 5 μM in (**e**–**h**).

**Figure 6:**
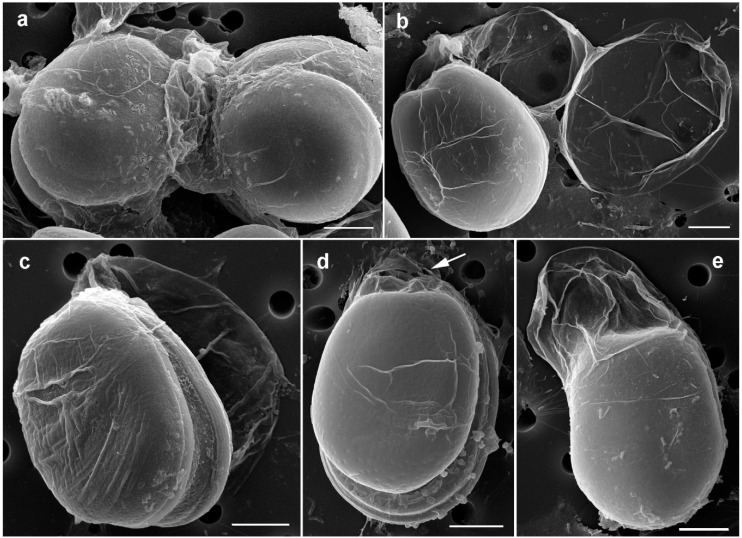
Scanning electron micrographs of *Prorocentrum malayense* BRM2 showing sheaths around the cells. (**a**) Several cells sticking together in mucus. (**b**) Note the empty sheaths. (**c**) Two cells with sheaths sticking together on top of an empty sheath. (**d**) Note the irregular mucous stalk-like structures at the apical cell end (arrow). (**e**) Cell leaving its skin-like sheath. Scale bars = 10 μM.

Description: Cells were broad to elongate oval, 36.9–43.7-μM long and 30.5–34.9-μM deep (*n* = 16) (Figure 4a–d and Figure 5a–c). The golden-brown chloroplasts had a central pyrenoid visible due to the starch sheath (appearing as a ring-like structure) (Figure 4a–c). The kidney-shaped nucleus was located posterior (Figure 4a,d). The periflagellar area was wide and V-shaped, slightly excavating the right thecal plate (Figure 4a,b and Figure 5a,b), and had no collar on the left thecal plate (Figure 5c). The intercalary bands were smooth (Figure 5d). Thecal plates were smooth (Figure 5a–e) with shallow depressions only around the pores (Figure 5e). Thecal pores of two size classes (Figure 5e) were regularly scattered over the thecal surface with the plate centre devoid of pores (Figure 5a–d), and pores become clearly denser close to the margin (Figure 5a–c). An irregular “marginal row” of pores was recognized (Figure 5d,e). Large pores were 0.22–0.24 μM, and small pores were 0.16–0.20 μM in diameter. Pores were encircled by a ring-like structure (Figure 5e). Nine platelets were present in the periflagellar area: 1a, b, 2, 3, 4, 5, 6, 7 and 8 (Figure 5f–h). Platelet 1a had no special ornamentation or structure. The second platelet seemed to have a large pore (Figure 5h). A special feature observed in this species was the production of irregular mucous stalk-like structures at the apical cell end (Figure 4a, e) and of sheaths around the cell (Figure 4b–d and Figure 6a–d). Cells were divided inside an irregular hyaline mucus cover (Figure 4f). What was conspicuous was that single cells left their skin-like sheath regularly (Figure 4g and Figure 6e), and disembodied remains of the sheath stayed behind and persisted for a longer time (Figure 4h and Figure 6b). This was only observed in culture. The cell extraction method did not allow for observing non-motile life cycle stages on the sand grains in the natural habitat.

### 2.2. Phylogenetics

**Figure 7:**
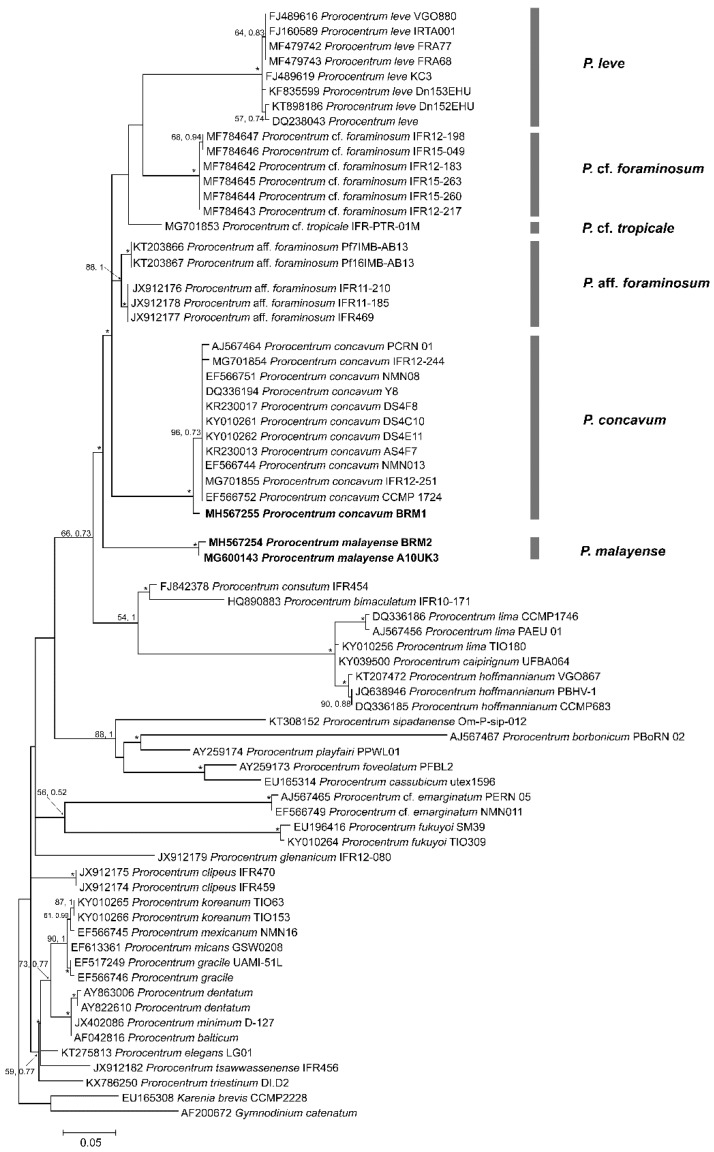
Maximum likelihood (ML) phylogenetic trees of *Prorocentrum* species/strains based on the D1–D3 region of LSU rDNA. Numbers at nodes represent posterior probabilities from Bayesian inference (BI) and bootstrap support values from maximum likelihood analysis based on 1000 pseudo-replicates. * represents 1 and 100 support values for BI and ML, respectively. Sequences obtained in this study are highlighted in bold.

The phylogenetic results for the *Prorocentrum* strains in the present study are shown in Figure 7. The G+C content of the partial D1–D3 LSU rDNA regions investigated in this study for BRM1 and BRM2 was 50.9% and 51.9%, respectively. Maximum likelihood (ML) and Bayesian inference (BI) analyses performed on the LSU rDNA regions of the two isolates with additional reference sequences from GenBank identified numerous strongly supported clades exhibiting a similar topology to previous studies. The BRM1 strain (Genbank accession number MH567255) was grouped with other *P. concavum* sequences retrieved from GenBank and was branched at the base of this species subclade. The BRM2 strain (Genbank accession number MH567254), *P. malayense*, formed a novel fully supported monophyletic lineage (BI = 1.00; ML = 100) at the base of the clade comprising *P. foraminosum*, *P.* aff. *foraminosum P. concavum, P.* cf. *tropicale* and *P. leve* (Figure 7). The genetic *p* distance between the *P. concavum* isolates, including BRM1, that was used in the phylogenetic analyses varied from 0.0–0.5% (Table 1). However, the *p* distance values between the closely related species in clade A varied from 12.9–16.4% (Table 1).

### 2.3. Toxin Analyses

No okadaic acid, dinophysistoxin-1 or dinophysistoxin-2 were detected from *P. malayense* and *P. concavum* cell pellets in the given culturing conditions at a detection limit of 0.05 pg cell^−1^. Analysis was included for esterified forms of the target biotoxins.

## 3. Discussion

### 3.1. Morphological Study

Studies on the diversity, evolution and ecology of benthic dinoflagellates are challenging due to a paucity of field data, under-sampling at a spatiotemporal scale, low abundance of certain species and difficulties in establishing and maintaining cultures. “Cryptic” species also appear to be common and further complicate the correct identification of dinoflagellate species [18]. Almost 70 *Prorocentrum* species have been described to date, of which about 30 species are associated with benthic habitats [12]. In this study, we revealed two benthic species of *Prorocentrum* from samples collected from a tidal flat in Broome, in north Western Australia, that were morphologically and phylogenetically identified as *P. concavum* and *P. malayense*.

Our field specimens and culture of *P. concavum* corresponded to the original description [39] of the species and further observations (e.g., References [13,14,19,39,40,41,42,43,44,45]) (see Supplementary Table S1, for details). Morton [41] described *P. faustiae* Morton, a species extremely similar to *P. concavum*, from reef flats near Heron Island, Great Barrier Reef, as broad oval cells 43–49 µm in length and 38–42 µm in width (synonymized with *P. concavum* by Chomérat et al. [13]), while Mohammad-Noor et al. [43] reported a range of 43–53 μM in length and 38–48 μM in width for *P. concavum* (but a larger range for specimens identified as *P.* cf. *faustiae* (see Supplementary Table S1, for details). *P. arabianum* Morton and Faust [44] was synonymized with *P. concavum* after a detailed reinvestigation by Mohammad-Noor et al. [19]. A group of several species similar to *P. concavum* was recognized by Hoppenrath et al. [12]. *Prorocentrum borbonicum* has significantly smaller cells with foveate ornamentation and pores inside and between depressions [26]. *Prorocentrum foraminosum* has a smooth to slightly foveate thecal surface and divides into hyaline cysts [12,46]. *Prorocentrum leve* cells have a smooth surface and can form chains within a hyaline envelope [34,47].

*Prorocentrum malayense* is very similar to *P. leve* and *P. foraminosum*, as it also has a smooth thecal surface and a similar pore pattern, but the Australian strain described in the present study produced mucous stalk-like structures at the apical cell end and sheaths around single cells. Chain formation, as has been observed in *P. leve* and the type strain of *P. malayense*, has never been observed in the Australian strain [5,34]. The platelet formula and platelet morphologies showed differences from *P. leve*, as *P. leve* normally has eight platelets, and sometimes platelet eight was split into two [47]. *Prorocentrum foraminosum* has differently shaped (narrower) platelets 2 and 3 [13,46]. Cells of *P. foraminosum* are more elongated (oblong), narrowing toward the periflagellar area, and are slightly larger (46–66 µM in length) compared to *P. malayense* [12]. A *Prorocentrum* species very similar to the Australian *P. malayense* strain, *Prorocentrum* sp. 1, has been described in southern Australia [4]. The thecal plates were smooth with shallow depressions containing pores, and the non-motile cells were always covered by a transparent mucoid layer [4]. Our description of *P. malayense* differs from the original description in that the production of irregular mucous stalk-like structures at the apical cell end (Figure 4a,e) and sheaths around the cell (Figure 4b–d and Figure 6a–d) were not originally described. Chain formation was not recorded. These were consistent features that were observed in cells taken from natural samples, and they were also found in *Prorocentrum* sp. 1, described from southern Australia [4]. In addition, platelets 4 and 5 differed in size and shape: platelet 4 (relatively small and narrow) was larger and wider, and platelet 5 (described as narrow and J-shaped) was wide in the strain investigated in this study (Figure 5f).

### 3.2. Phylogenetic Analysis

In this study, we used the D1–D3 domains of the large ribosomal subunit rRNA (LSU) gene for phylogenetic analysis. LSU rDNA fragments can often resolve close species relationships between benthic dinoflagellates compared to other ribosomal markers, such as small-subunit (SSU or 18S) rDNA and internally transcribed spacer (ITS) regions, and they are extensively used, in conjugation with microscopy, for phylogeographic studies of benthic genera such as *Coolia, Gambierdiscus, Ostreopsis*, *Prorocentrum* and *Thecadinium* [13,18,20,48,49,50,51]. Genetic distance estimates among *Prorocentrum* species using the LSU rDNA region have been crucial in species-level discrimination and have previously been used to report conspecific species, as in the case of *P. arenarium* and *P. lima* [19,42]. Our study reports a divergence varying from 12.9–16.7% between *P. malayense* and other species of Clade A *sensu* Chomérat et al. [13] (Table 1). *P*. aff. *foraminosum*, which was initially identified as *P. foraminousm* in the temperate Atlantic and North Sea, diverged by 12.9%, whereas *P.* cf*. foraminosum* strains that were recently identified in the Caribbean by Chomérat et al. [13] diverged by 16.4% from *P. malayense* (Table 1). A genetic distance of 9.3% was recovered between the two “*P. foraminosum*” subclades, suggesting that a detailed analysis of the European “*P. foraminosum*” isolates is required, as they may constitute a new species [13]. A study by Zhang et al. [52] reported five *P. lima* “morphotypes” and associated ITS and LSU rDNA sequences with them. Two “morphotypes” were defined based on one strain; therefore, the morphological diversity associated with this genotype and its overlap with other morphotypes could not be assessed. Previous information, including ITS sequences and the morphology of *P. lima* genotypes, did not show a consistent difference [53]. A recent study named the new species *P. caipirignum* (earlier identified as *P. lima* morphotype 4), and it was less than 2% genetically distinct from the *P. hoffmannianum* species complex clade based on an LSU rDNA gene fragment [36]. *P. belizeanum* and *P. hoffmannianum* were found to be con-specific, as they had a less than 0.56% genetic distance divergence between geographically isolated populations [16]. Such low intraspecific genetic differences (between 0.0–0.5%) between *P. concavum* isolates were also observed in our study (Table 1).

Previously, Mohammad-Noor et al. [19] reported a sequence divergence of 0.2–19.8% in LSU rDNA fragments between *Prorocentrum* species from clade A, which was a large clade that included *P. concavum*, and Clade B, which included the *Prorocentrum lima* complex. Our results, including the new species *P. malayense*, extended that divergence between the two clades to 21.1–24.3% (see Supplementary Table S2, for details). Kohli et al. [6] used an eDNA molecular barcoding approach based on short cytochrome B (*cob*) and SSU marker regions to identify benthic dinoflagellates from several sites in Western Australia, including Town Beach (sample collection site for this study). A total of 4682 *cob* sequences were obtained, of which 310 were identified as Prorocentrales: *P. concavum* was identified as present, but was not further described [6]. A number of unique sequences were identified as being related to *P. concavum*, suggesting that those OTUs may have been *P. malayense*, which was not described at the time.

### 3.3. Toxicity

Previously, OA has been reported in *P. concavum* [25,54], and DTX-1 has been reported in *P*. aff. *foraminosum* (as *P. foraminosum*) [55,56]. However, no detectable amounts of OA or DTXs were found in the *P. malayense* and *P. concavum* strains in this study. Previously, *P. concavum* from Japanese waters have demonstrated high ichthyotoxic bioactivity, but did not produce any detectable OA [8]. In addition, *P. concavum* from the Gulf of Oman (as *P. arabianum*) produced a cytotoxic and ichthyotoxic compound, but OA was not observed [44]. Recently, Luo et al. [45] also did not find detectable amounts of OA from *P. concavum* isolates from the South China Sea. Such results could have been due to strain-specific variations in the toxin production of *P. concavum* isolates, since these previous results were obtained from the investigation of a single strain or only a few strains. In addition, culturing conditions are known to play a vital role in the toxin production of dinoflagellates and should be considered while investigating the toxin production of *P. concavum* and related species. Such studies highlight that *Prorocentrum* species could possibly produce rare toxic molecules that are not detected by standard LC–MS/MS monitoring analyses, and hence a detailed non-targeted analysis of toxic compounds from the two species requires further investigation.

The genus *Prorocentrum* has not been studied much in Australian waters, despite recurrent occurrences from temperate and tropical locations [6,15,41,57]. As emphasized in recent studies, there is a serious need for a more comprehensive study on this genus in order to clarify the species delimitations for several taxa [58]. In this study, we established the first reports of *P. malayense* and *P. concavum* from tropical north Western Australia. Increasing knowledge about the species identity, distribution and toxicity of *Prorocentrum* spp. will enable more effective monitoring of harmful algal taxa in Australian waters.

## 4. Materials and Methods

### 4.1. Sampling and Culture Establishment

Sandy intertidal sediments without discoloration were collected from the surface of the tidal flat during low tide in Town Beach, Broome (17°97’S, 122°23’E) during May 2011 (Figure 1). The first 3–5-cm layer of the sand/silt layer was collected using a flat spoon, as described previously in Murray and Patterson [59]. At the time of collection, the sea surface temperature was 22 °C, and the salinity ranged from 38 to 40. The organisms were isolated according to the Uhlig method [5,60]. In summary, the samples were placed in plastic tubes with their bottom opening sealed with a strained gauze to avoid any larger fauna or debris passing through. A Petri dish was placed under the tube, and frozen seawater was added, creating a temperature and salinity gradient and making the organisms migrate down into the Petri dish. The separated organisms were isolated using a micropipette in local filtered seawater and subsequently enriched with f/2 media at a salinity of 35 [61]: they were observed using a Leica DMIL inverted light microscope (Leica Microsystems GmbH, Wetzlar, Germany).

After several weeks, certain parts of the dish were covered with cells producing sheaths. Free-swimming cells were separated from sheath-producing cells, thereby aiding in the establishment of two monoclonal non-axenic *Prorocentrum* cultures. The cultures were maintained at Senckenberg am Meer, the German Centre for Marine Biodiversity Research (DZMB). Both cultures were maintained at 19 °C under a photo-illumination of 60–80 μmol m^−2^ s^−1^ and a 12:12-h light–dark cycle.

### 4.2. Microscopy

Living cells were picked using a Leica DMIL inverted microscope (Leica Microsystems GmbH, Wetzlar, Germany) placed on an object slide, and observed with a Leica DMRB fluorescence microscope equipped with differential interference contrast optics at a 400- and 640-times magnification with oil immersion objectives. Digital photos were taken using a Leica DFC290 and DFC420C camera.

For SEM, the cultures were fixed with Lugol’s solution and stored in the dark. Some fixed subsamples were treated with 37% H_2_O_2_ for three to four days to digest the membranes and mucilage on top of the cells (to “clean” the surface) through oxidative processes. Cells were placed on a 5-µM Millipore filter, rinsed in distilled water and dehydrated in a series of increasing ethanol concentrations (30%, 50%, 70%, 85%, 90%, 100%), followed by chemical drying with hexamethyldisilazane (HMDS) at room temperature for 20 min and finally at 50 °C in a drying oven for 5 min. The sample/filter was mounted on a stub and sputter coated with gold palladium (Bal-Tec SCD 050; BAL-TEC Präparationsgerätevertrieb, Wallof, Germany). Cells were observed using a Hitachi S-3200N (Tokyo, Japan) and a Tescan VEGA3 (Elekronen-Optik-Service GmbH, Dortmund, Germany) microscope at 20 and 15 kV using a secondary electron detector. The terminology of cell orientation, designation of thecal plates and platelets and ornamentation followed Hoppenrath et al. [12].

### 4.3. DNA Extraction and PCR Amplification

Genomic DNA of the new species was extracted from 10 living cells of the new species using a MasterPure Complete DNA and RNA Purification Kit (EPICENTRE, Madison, WI, USA) according to the manufacturer’s protocol. Air-dried DNA samples were shipped to the University of Technology Sydney, where they were resuspended in 20 μL of 1 × TE (Tris-EDTA, pH 8) buffer. Fifty mL of dense *P. concavum* culture was centrifuged at 1500× *g* for 5 min at room temperature, and DNA was extracted from the algal pellet using a modified CTAB (hexadecyltrimethylammonium bromide) buffer recipe as previously described [18,62]. DNA from both strains was quantified using a NanoDrop ND-1000 (NanoDrop Technologies, Wilmington, DE, USA) prior to PCR.

The partial nuclear LSU rRNA gene region D1–D3 was amplified in 25 μL of reaction volume containing 12.5 μL of 2× Immomix (Bioline, Sydney, Australia), 10 pmol of forward D1R (5’-ACC CGC TGA ATT TAA GCA TA-3’) [63] and reverse D3B (5’-TCG GAG GGA ACC AGC TAC TA-3’) [64], 1 μg μL^−1^ of Bovine Serum Albumin (BSA) (Biolabs, Arundel, Australia), 1 μL of template DNA and molecular-grade water to give the final volume. Thermocycling conditions consisted of an initial denaturing step of 95 °C for 10 min followed by 35 cycles of 95 °C for 20 s, 57 °C for 30 s of annealing, 72 °C for 1 min and a final extension of 72 °C for 7 min. PCR products were purified with DNA Clean and Concentrator (Zymo Research, Irvine, CA, USA) according to the manufacturer’s protocol. The PCR products were sequenced using a commercial service (Macrogen Inc., Seoul, Korea). The forward and reverse sequences were trimmed, aligned and visually refined using Geneious v7 (Newark, NJ, USA) [65] and were deposited in GenBank.

### 4.4. Phylogenetic Analysis

Phylogenetic analysis was performed (using both maximum likelihood (ML) and Bayesian inference (BI) approaches) on the sequences acquired from the two species. Sixty-six LSU rDNA sequences from other *Prorocentrum* species (see Supplementary Table S3, for details), along with sequences from *Karenia brevis* CCMP2228 (EU165308) and *Gymnodinium catenatum* (AF200672) as outgroups, were aligned with the two sequences. Multiple sequence alignments were performed using the ClustalW v1.6 program as implemented in MEGA v6 [66], followed by manual inspection. For the ML tree, a substitution model was selected using jmodeltest 2 [67] based on the lowest Bayesian information criterion as a measure of the relative quality of the model. An ML tree was generated in MEGA v6 using general time-reversible (GTR) + G (gamma) + I (inversions) with a 5 G category substitution model for the sequence analysis. Nodal support of the ML tree was estimated via a bootstrap algorithm with 1000 replications. The final dataset was comprised of 70 LSU rDNA sequences and 1577 sites (including gaps), of which 637 were conserved, 929 were variable and 690 were parsimony sites. Bayesian analysis was performed using MrBayes v3.2 [68] as implemented in Geneious v7 using GTR + G model. Four independent Markov Chain Monte Carlo simulations were run simultaneously for 2,000,000 generations. Trees were sampled every 1000 generations, and 1000 trees were discarded as burn-in (log-likelihood stabilization). The phylogenetic tree was represented using the ML results and bootstrap values (greater than 50) from the analysis and posterior probability values (greater than 0.5) from the bayesian analysis. The uncorrected genetic distance (*p*) between different *Prorocentrum* species was analysed using a *p*-distance model and bootstrap procedure (1000 replicates) in MEGA v6. All positions containing gaps and missing data were eliminated for the analysis, and *p*-distances between all strains and species used in the analysis are displayed in the Supplementary Table S2.

### 4.5. Determination of Okadaic Acid (OA) and Dinophysistoxins (DTXs)

Cell pellets of the new species (approx. 2 × 10^3^ cells) and *P. concavum* (approx. 4 × 10^5^ cells) were dried and shipped to the Cawthron Institute, New Zealand, where they were subsequently screened for the presence of free and esterified forms of OA and dinophysistoxin-1 and -2 (DTX-1 and DTX-2). Analyses were performed using a quantitative LC–MS/MS method developed at the Cawthron Institute (as described in McNabb et al. [69]) and used routinely for the analysis of these biotoxins in shellfish. In summary, each sample was extracted with 2 mL of 90% aqueous methanol using ultrasonication (59 Hz for 10 min). Cellular debris was pelleted using centrifugation (3200× *g* for 5 min at 4 °C), and the supernatant was decanted into a separate tube. A second extraction was performed using identical conditions. Both supernatants were pooled and mixed, and a 1-mL aliquot was transferred to a glass autosampler vial for the analysis of free OA, DTX-1 and DTX-2. A second 0.5-mL aliquot was hydrolysed using 62.5 µL of 2.5-M sodium hydroxide (heated at 76 °C for 45 min), neutralised with 62.5 µL of 2.5-M acetic acid, cooled in an ice slurry and finally centrifuged (17,000× *g* for 2 min) to afford a clear supernatant. This base hydrolysis process liberates esterified forms of the toxins, if present. A 300-µL aliquot was transferred into a glass autosampler vial and analysed for total OA, DTX-1 and DTX-2 content. LC–MS/MS analysis was carried out on a waters Acquity UPLC i-Class system coupled to a Waters Xevo TQ-S triple quadrupole mass spectrometer with electrospray ionisation. Chromatographic separation was achieved using a BEH Shield RP18 column (Waters 50 × 2.1 mm; 1.7 µm) using the mobile phases; A) acetonitrile−Milli-Q (5:95, v/v), and B) acetonitrile−Milli-Q (95:5, v/v), each with 50 mM formic acid and 2.53 mM ammonium hydroxide. A flow rate of 0.5 mL min^−1^ was used with an initial solvent composition of 100% A with a step gradient to 80% B over 5 min. For column washing, the B solvent was increased to 100% and held for 2.5 min before being returned to the initial conditions of 100% A for re-equilibration, giving a total run time of 8 min. Using these gradient conditions, the OA-group toxins eluted for between 3 and 4 min. For mass spectrometry analysis, multiple reaction monitoring (MRM) transitions were established for the [M + Na]^+^ ions of the various toxins in +ESI (Electrospray ionization) mode (827.5 > 723.4 OA/DTX-2; 841.5 > 737.4 DTX1) and for [M–H]^−^ in –ESI mode (803.5 > 255.2 OA/DTX-2; 817.5 > 255.2 DTX1). Data acquisition and processing was performed using TargetLynx software (Waters-Micromass, Manchester, UK), and the limit of detection (LoD) for all analytes was determined to be 0.05 pg cell^−1^.

## Figures and Tables

**Table 1:** Mean genetic distance (pairwise uncorrected *p*-distances) within and net-between species closely related to *P. concavum* and *P. malayense* based on Clustal W alignment of 34 D1–D3 LSU rDNA sequences from the phylogenetic analysis. Standard error estimate(s) are shown in brackets and were obtained through a bootstrap procedure (1000 replicates).

Species	*P*. *concavum*	*P*. cf. *foraminosum*	*P*. aff. *foraminosum*	*P*. *leve*	*P*. cf. *tropicale*	*P*. *malayense*
***P*. *concavum*** **(*n* = 12)**	0.005 (0.002)					
***P*. cf. *foraminosum*** **(*n* = 6)**	0.158 (0.017)	0.001 (0.001)				
***P*. aff. *foraminosum*** **(*n* = 5)**	0.119 (0.015)	0.093 (0.013)	0.013 (0.004)			
***P*. *leve*** **(*n* = 8)**	0.181 (0.019)	0.153 (0.018)	0.138 (0.016)	0.005 (0.002)		
***P*. cf. *tropicale*** **(*n* = 1)**	0.132 (0.017)	0.103 (0.015)	0.084 (0.013)	0.151 (0.017)	NA	
***P*. *malayense*** **(*n* = 2)**	0.163 (0.018)	0.164 (0.018)	0.129 (0.016)	0.167 (0.018)	0.159 (0.018)	0.015 (0.004)

NA represents not available; *n* represents the number of sequences from each species used in the analysis.

## Supplementary Materials

The following are available online at https://www.mdpi.com/2072-6651/11/10/571/s1, Table S1: Morphological reports of *Prorocentrum concavum* and its previously used synonyms; Table S2: Mean genetic distance (pairwise uncorrected *p*-distances) between 70 strains/species of *Prorocentrum* species and outgroups based on Clustal W alignment of D1-D3 LSU rDNA sequences from the phylogenetic analysis. Standard error estimates are shown in blue and were obtained through a bootstrap procedure (1000 replicates); Table S3: List of *Prorocentrum* spp. used for phylogenetic reconstruction (sequences obtained from Genbank).
