# Supplementary material for: Morphology and Phylogenetics of Benthic Prorocentrum Species (Dinophyceae) from Tropical Northwestern Australia
Source: Toxins (Basel). 2019 Sep 30;11(10):571. doi: 10.3390/toxins11100571 (PMC6833055; doi:10.3390/toxins11100571)
Supplement: Supplementary file 1 [file toxins-11-00571-s001.zip › toxins-581213 Supplementary table s1.docx]

Supplementary Materials: Morphology and Phylogenetics of Benthic Prorocentrum Species (Dinophyceae) from Tropical North-Western Australia

Arjun Verma, Aniuska Kazandjian, Chowdhury Sarowar, D. Tim Harwood, J. Sam Murray, Insa Pargmann, Mona Hoppenrath and Shauna A. Murray

**Table S1.** Morphological reports of *Prorocentrum concavum* and its previously used synonyms.

| **S. No.** | **Species Name** | **Length (in µM)** | **Depth (in µM)** | **Location** | **Pores (in µM)** | **Reference** |
| --- | --- | --- | --- | --- | --- | --- |
| 1. | *P. concavum* | 44–45 | 40 | French Polynesia, New Caledonia, Ryukyu Islands | NA | [39] |
| 2. | *P. concavum* | 50–55 | 38.0–45.0 | Twin Cays, Belize | NA | [40] |
|  | *P. concavum*  (syn. *P. faustiae*) | 43.0–49.0 | 38.0–42.0 | Heron Island, Australia | 0.1–0.2 | [41] |
| 3. | *P. concavum* PPAN04 | 58.0–60.0 | 50.0–52.0 | Contadora Island, Gulf of Panama | NA | [42] |
| 4. | *P. concavum* CCMP1724 (syn. *P. arabianum*) | 42–48 | 35–40 | Gulf of Oman | 0.14 | [43] |
| 5. | *P. concavum* | 43.0–53.0 | 38.0–48.0 | Malaysian peninsula | 0.12–0.23 | [44] |
| 6. | *P.* cf. *faustiae* | 45.0–60.0 | 38.0–53.0 | Malaysian peninsula | 0.10–0.20 | [44] |
| 7. | *P. concavum* CCMP 1724 (re-examined) | 38–49 | 35 40 | Gulf of Oman | Large: 0.17–0.21 Small: 0.1–0.13 | [19] |
| 8. | *P. concavum* | 45.7–50.2 | 37.7–42.4 | South China Sea | 0.13–0.27 | [45] |
| 9. | *P. concavum* | 46.0–48.0 | 38.0–44.0 | Martinique Island, Caribbean Sea | 0.25–0.30 | [13] |
| 10. | *P. concavum* SS1201, SP001 | 43.2–51.3 | 38.2–48.2 | Perhentian Islands, Malaysia | Large: 0.15–0.8 Small: 0–0.15 | [14] |
| 11. | *P. concavum*BRM1 | 40.6–50.2 | 36.2–46.3 | Broome, Western Australia | Large: 0.15–0.25 Small: 0.05–0.10 | This study |

NA represents not available.
